# Genetic and Metabolite Variability in One-Carbon Metabolism Applied to an Insulin Resistance Model in Patients With Schizophrenia Receiving Atypical Antipsychotics

**DOI:** 10.3389/fpsyt.2021.623143

**Published:** 2021-05-25

**Authors:** Kristen M. Ward, Kyle Burghardt, A. Zarina Kraal, Andrew Jaeger, Larisa Yeomans, Cora McHugh, Alla Karnovsky, Kathleen A. Stringer, Vicki L. Ellingrod

**Affiliations:** ^1^Department of Clinical Pharmacy, College of Pharmacy, University of Michigan, Ann Arbor, MI, United States; ^2^Department of Pharmacy Practice, Eugene Applebaum College of Pharmacy and Health Sciences, Wayne State University, Detroit, MI, United States; ^3^Psychology Department, College of Literature, Science, and the Arts, University of Michigan, Ann Arbor, MI, United States; ^4^Nuclear Magnetic Resonance (NMR) Metabolomics Laboratory, College of Pharmacy, University of Michigan, Ann Arbor, MI, United States; ^5^Department of Computational Medicine and Bioinformatics, School of Medicine, University of Michigan, Ann Arbor, MI, United States; ^6^Michigan Regional Comprehensive Metabolomics Resource Core, University of Michigan, Ann Arbor, MI, United States; ^7^Division of Pulmonary and Critical Care Medicine, School of Medicine, University of Michigan, Ann Arbor, MI, United States; ^8^Department of Psychiatry, School of Medicine, University of Michigan, Ann Arbor, MI, United States

**Keywords:** pharmacogenomics, metabolomics, folate, one-carbon metabolism, cardiovascular disease, antipsychotics, insulin resistance

## Abstract

**Background:** Patients with schizophrenia are at high risk of pre-mature mortality due to cardiovascular disease (CVD). Our group has completed studies in pharmacogenomics and metabolomics that have independently identified perturbations in one-carbon metabolism as associated with risk factors for CVD in this patient population. Therefore, this study aimed to use genetic and metabolomic data to determine the relationship between folate pharmacogenomics, one-carbon metabolites, and insulin resistance as measured using the homeostatic model assessment for insulin resistance (HOMA-IR) as a marker of CVD.

**Methods:** Participants in this pilot analysis were on a stable atypical antipsychotic regimen for at least 6 months, with no diabetes diagnosis or use of antidiabetic medications. Participant samples were genotyped for *MTHFR* variants rs1801131 (*MTHFR* A1298C) and rs1801133 (*MTHFR* C677T). Serum metabolite concentrations were obtained with NMR. A least squares regression model was used to predict log(HOMA-IR) values based on the following independent variables: serum glutamate, glycine, betaine, serine, and threonine concentrations, and carrier status of the variant alleles for the selected genotypes.

**Results:** A total of 67 participants were included, with a median age of 47 years old (IQR 42–52), 39% were female, and the median BMI was 30.3 (IQR 26.3–37.1). Overall, the model demonstrated an ability to predict log(HOMA-IR) values with an adjusted *R*^2^ of 0.44 and a *p*-value of < 0.001. Glutamate, threonine, and carrier status of the *MTHFR* 1298 C or *MTHFR* 677 T allele were positively correlated with log(HOMA-IR), whereas glycine, serine, and betaine concentrations trended inversely with log(HOMA-IR). All factors included in this final model were considered as having a possible effect on predicting log(HOMA-IR) as measured with a *p*-value < 0.1.

**Conclusions:** Presence of pharmacogenomic variants that decrease the functional capacity of the MTHFR enzyme are associated with increased risk for cardiovascular disease, as measured in this instance by log(HOMA-IR). Furthermore, serine, glycine, and betaine concentrations trended inversely with HOMA-IR, suggesting that increased presence of methyl-donating groups is associated with lower measures of insulin resistance. Ultimately, these results will need to be replicated in a significantly larger population.

## Introduction

The rate of mortality due to cardiovascular disease (CVD) is 2–3 times higher in patients with severe mental illness, such as schizophrenia and bipolar disorder, when compared to the general adult population (1, 2). Among risk factors for CVD, insulin resistance is important as an early, defining feature of diabetes and metabolic syndrome (3, 4). Meta-analyses have demonstrated that diabetes and metabolic syndrome occur at higher rates in patients with schizophrenia, and that these rates are higher yet among patients exposed to antipsychotic drug therapy (5, 6). However, the mechanisms underlying increased rates of insulin resistance and CVD are poorly understood and likely due to a complex interplay of genetic and environmental influence such as medication use and cigarette smoking.

**Figure 1:**
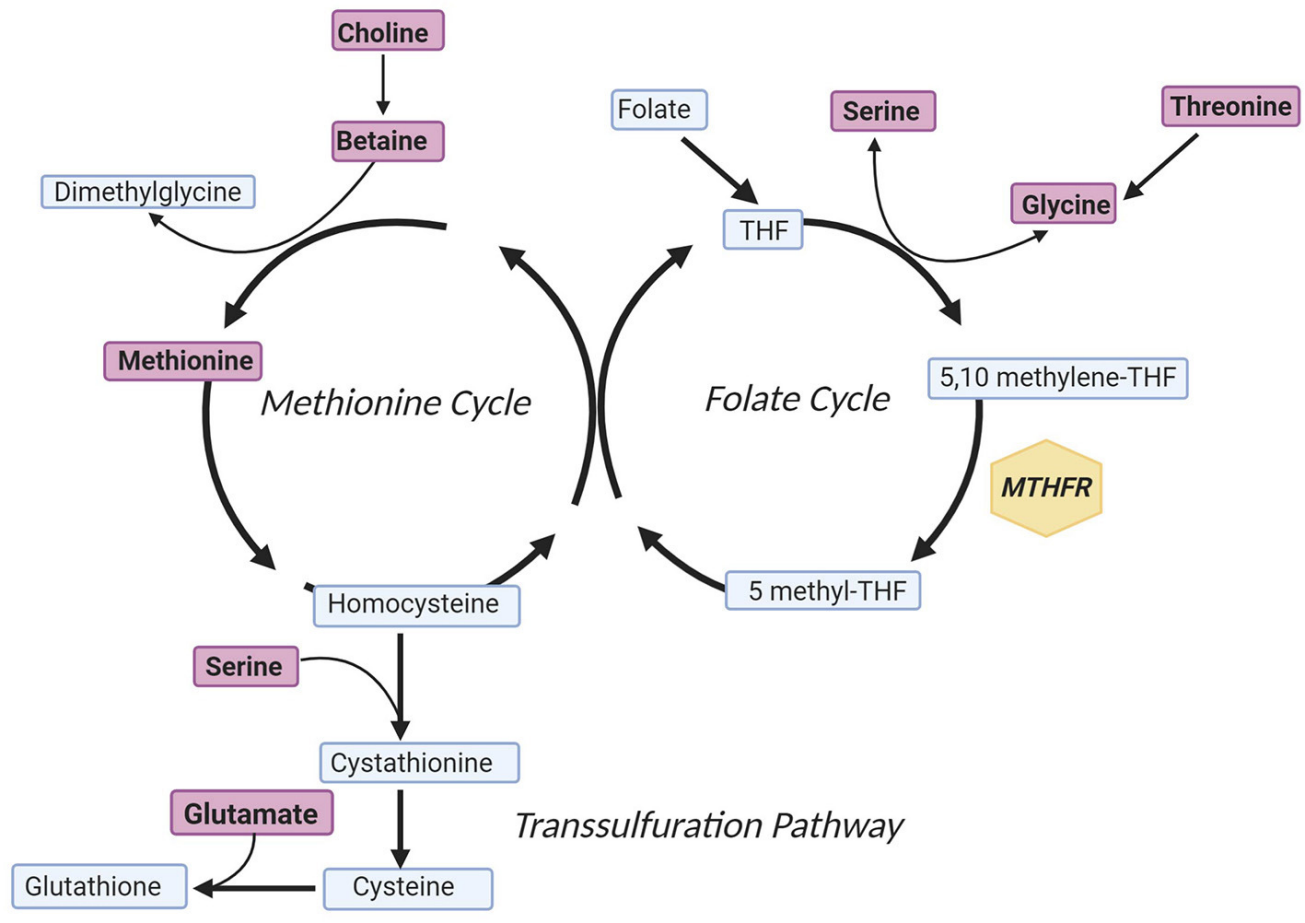
Simplified One-Carbon Metabolism. Metabolites included in the model predicting log(HOMA-IR) concentration are indicated in purple, and the gene of interest is annotated within a yellow hexagon. In brief, the methionine cycle is involved in methyl-transferase reactions, the folate cycle is involved in purine synthesis, and the transsulfuration pathway culminates with formation of the antioxidant glutathione (9). The figure was created with Biorender.com. MTHFR, methylenetetrahydrofolate reductase; THF, tetrahydrofolate.

One potential mechanism of CVD in patients with schizophrenia is aberrant one-carbon metabolism (7). One-carbon metabolism includes a network of pathways such as the folate cycle, methionine cycle, and the transsulfuration pathway that are involved in critical processes such as methylation, DNA synthesis, and protein synthesis (8). These pathways include essential one-carbon metabolite sources and products such as serine, choline, betaine, threonine, glutamate, and methionine (Figure 1). Additionally, within the folate cycle, genetic variants in the gene coding for the methylenetetrahydrofolate reductase (MTHFR) enzyme have been given particular attention based on studies associating carrier status with increased rates of CVD risk factors, such as metabolic syndrome (10–12). This enzyme plays an important role in the folate cycle by converting dietary folate to the active form, L-methylfolate (5 methyl-THF; Figure 1) (13). The functional impact of decreased MTHFR activity is accumulation of homocysteine and decreased synthesis of neurotransmitters (14, 15). Two genetic variants in the gene coding for the MTHFR enzyme that have been associated with decreased enzyme activity are *MTHFR* C677T and *MTHFR* A1298C. The presence of either variant leads to amino acid base pair changes that ultimately reduce enzyme activity, and variant carrier status has been linked to risk of metabolic syndrome in patients treated with antipsychotics (11, 16–19). Variants within the *MTHFR* gene have also been studied in patients without serious mental illness, and associations with variant carrier status and inflammation, adverse metabolic outcomes, and cardiovascular events have been inconsistent and in some studies dependent on population, age, or folate serum levels (20–22).

Metabolomics is the science of small molecule profiling in biological samples, and in relation to the other “omics” approaches, it reflects downstream activities of the genome, transcriptome, and proteome (23–25). This makes metabolomics useful for identifying mechanisms of treatment and disease. In an NMR metabolomics experiment, our group identified that metabolites attributable to one-carbon metabolism differentiated patients with schizophrenia on antipsychotics based on fasting insulin concentration (26). Ultimately, our group has completed genetic and metabolomics analyses that have independently identified an association between variable one-carbon metabolism and CVD risk when identified as metabolic syndrome and variable fasting insulin (10, 11, 26). Here, we expand upon these findings by combining metabolomics and genomics to predict CVD risk using the homeostatic model assessment of insulin resistance (HOMA-IR) as a surrogate CVD risk measure (27). HOMA-IR has been studied extensively as a minimally invasive, clinically accessible method to assess insulin resistance and beta-cell function that correlates well with values obtained using the hyperinsulinemic euglycemic clamp technique (28).

Therefore, the aim of this study was to determine the extent to which genetic variants in the *MTHFR* gene and metabolites important in one-carbon metabolism could predict insulin resistance in patients with schizophrenia that were not diagnosed with diabetes or treated with diabetes medications. We hypothesized that the aforementioned metabolites and *MTHFR* variant carrier statuses would contribute to a model that would be able to predict log(HOMA-IR) concentration as determined by a *p*-value of < 0.05.

## Methods

### Participants

Participants included in this analysis were selected from a large, observational, cross-sectional study investigating CVD in patients diagnosed with a schizophrenia spectrum disorder or bipolar disorder per the Diagnostic and Statistical Manual of Mental Disorders, Fourth Edition (29). The parent study's inclusion criteria included use of an antipsychotic for at least 6 months prior to enrollment, with no antipsychotic regimen changes within the previous 8 weeks. Additional inclusion criteria were ages between 18 and 90 years old and no diabetes diagnosis prior to starting an antipsychotic. To be included in this analysis participants were restricted to those using an atypical antipsychotic for the treatment of a schizophrenia spectrum disorder, and no diagnosis of diabetes or use of medications that impact blood glucose regulation, such as metformin. Furthermore, subjects had to have had prior metabolomics profiling as part of a previous investigation (26). Atypical antipsychotic chlorpromazine equivalents were calculated using methods previously described by Andreasen et al. (30) and Woods (31).

Demographics, clinical histories, fasting blood draws and anthropometric measurements were performed at the University of Michigan's Clinical Research Unit (MCRU; http://www.michr.umich.edu/services/mcru). Members of the study team collected data from participants on the course of their disease and treatment (for example time since diagnosis and antipsychotic medication trials). HOMA-IR was calculated according to Matthews et al. (27) utilizing fasting insulin and fasting glucose values. The study protocol received Institutional Review Board (IRB) approval from the University of Michigan (IRBMED HUM00017774) and the following local organizations: the Detroit-Wayne County Community Mental Health Agency (DWCCMHA), Washtenaw County Health Organization (WCHO), and the Ann Arbor Veterans Affairs Medical Center. All subjects gave written informed consent in accordance with the Declaration of Helsinki.

### Genotyping

A salt precipitation technique was used to extract DNA from fasting whole blood samples (32). The variants analyzed were two single nucleotide polymorphisms (SNPs) in the gene coding for the methyltetrahydrofolate reductase (MTHFR) enzyme: (rs1801131/*MTHFR* A1298C and rs1801133/*MTHFR* C677T). For both variants, Assay Design 2.0 software with Pyrosequencing™ Technology (Qiagen, Valencia, CA) was utilized for primer design for polymerase chain reaction (PCR) primers and pyrosequencing sequencing primers as previously described (17). Briefly, PCR was used to amplify short segments of DNA containing the SNPs of interest with Thermo Scientific™ TaqMan™ Master Mix. After visualization of the PCR product using a 1.2% agarose gel with ethidium bromide or GelRed® (Biotium) dye, the PCR product was then used for genetic sequencing with a PyroMark MD sequencer with Qiagen pyrosequencing reagents (Qiagen, Valencia, CA) following their recommended protocols. In any instance of uncertain genotype assignment, the sequencing was repeated. When a sequencing genotype could not be identified conclusively for any given variant, it was excluded from the analysis. Pyrosequencing, as a method of genotyping, has been described in detail previously (33).

### Metabolomics

We obtained quantified metabolomics data from a previously completed study (26). Briefly, these data were generated from fasting (10 h) blood samples that were extracted into hydrophilic and hydrophobic metabolite fractions. The hydrophilic fraction was lyophilized and then re-suspended in deuterated water, with the addition of the internal standard formate, prior to NMR analysis by the University of Michigan's NMR Biochemical Core Laboratory. Among all identified metabolites in the previously completed study, only those involved in one-carbon metabolism were included in this analysis. When the samples were thawed for the metabolomics analysis, a small fraction was delivered to the Michigan Diabetes Research Center (http://diabetesresearch.med.umich.edu/) for fasting insulin and fasting glucose quantification in order to compute HOMA-IR.

### Statistical Analysis

Statistical analyses were completed with JMP Pro® 14.2.0 (SAS Institute Inc., Cary, NC). Demographic data were described as the mean and standard distribution, medians and interquartile range, or percentages of the study cohort, as appropriate. HOMA-IR was log transformed to address heteroscedasticity in the original model residual plot and its observed deviation from normality. Least squares regression was used to predict log(HOMA-IR) concentration as a function of the following quantified one-carbon metabolites: serine, choline, betaine, threonine, methionine, glutamate, and variant carrier status. *MTHFR* C677T TT or A1298C CC carriers were not considered separately in the model due to small numbers (3 and 5 participants for each genotype, respectively).

Ultimately, we proceeded with two models utilizing genomic and metabolomics factors to predict log(HOMA-IR). The first model included all the genetic and metabolite variables above followed by a second model only including variables with a possible effect (*p* < 0.1). Corrected Aikaike's Information Criterion (AICc) values were provided to compare models, and a model *p-*value < 0.05 was considered statistically significant (34).

Of note, a number of potential confounding variables, specifically age, BMI, race (simplified as Caucasian and non-Caucasian), smoking status, and chlorpromazine equivalents were added to the final regression model to assess for independent variable contribution from non-metabolomic and genomic factors. This was also done for use of clozapine or olanzapine when considering that these medications are known to be associated with a higher risk of adverse metabolic events (35).

## Results

### Participant Demographics

Participant demographic details are provided in Table 1. Of the 67 included participants, ~28% were currently treated with antipsychotics considered to have the highest risk of weight gain and glucose disturbance (i.e., olanzapine and clozapine) and the average CPZ equivalents was 558 mg in our sample. Despite the parent study allowing for enrollment of patients up to 90 years of age, the oldest participant in this secondary analysis was 60 years old. Finally, there was no significant departure from Hardy-Weinberg equilibrium for either *MTHFR* variant (*p* > 0.05).

**Table 1:** Participant demographics.

**Participant demographics (*N* = 67)**	
Age (IQR)	47 (42–52)
Sex (% female)	38.8
BMI (IQR)	30.3 (26.3–37.1)
Fasting insulin μU/mL (IQR)	16.6 (10.6–26.4)
Fasting glucose mg/dL (SD)	94.6 (12.0)
Log(HOMA-IR) (SD)	0.63 (0.29)
Use of clozapine or olanzapine (% yes)	28.4
MTHFR 677 T carrier (%)	28.8
MTHFR 1298 C carrier (%)	48.5
Race (% non-caucasian)	41.8
Smoker (% yes)	53.7
CPZ equivalents, AAP only in mg (IQR)	558.0 (367.0–727.2)
Total antipsychotic medication trials (IQR)	5 (3–6)
Atypical antipsychotic medication trials (IQR)	2 (2–4)
Typical antipsychotic medication trials (IQR)	2 (1–3)
Time since diagnosis in years (IQR)	19.0 (14.0–30.0)

*For continuous data, demographics were provided as means and standard deviations (SD) when values were normally distributed, otherwise as medians and interquartile ranges (IQR). CPZ, chlorpromazine; AAP, atypical antipsychotic*.

### Regression Analyses

Results of the least squares regressions analyses are provided in Tables 2 and 3. In Table 2, it is apparent that two metabolites within the one-carbon cycle (choline and methionine) do not significantly contribute to the prediction of log(HOMA-IR) despite a significant overall model (*p* = 0.001). These metabolites were removed from the analysis, and the final parameters are shown in Table 3. Decreasing the number of predictive factors improved the AICc, from 13.15 to 7.52 and the model remained significant (*p* = 1.622 E-5). With our participant sample size of 67, this allows for a power level of 0.8, assuming an effect size of 0.25 and an α of 0.05. Furthermore, the model remains significant when applying a Bonferroni correction as the *p-*value is below 0.007. For the two *MTHFR* SNPs, carrier status (i.e., carriers for reduced function enzymes) was associated with an increased log(HOMA-IR) value. In an exploratory analysis not shown here, when including the interaction of variant carrier status of the two *MTHFR* SNPs in the final model (described in Table 3) the interacting factor was not a significant contributor to predicting log(HOMA-IR) (*p*-value for the factor was 0.60).

**Table 2:** Least squares regression model parameters for initial input including all detected metabolites within one-carbon metabolism.

**Term**	**Estimate**	**Std. error**	**Prob > ItI**
**Regression model 1 (*****N*** **=** **67)**			
*MTHFR* 677 T Carrier	−0.06627	0.035263	0.07
*MTHFR* 1298 C Carrier	−0.07146	0.03099	0.03
Glycine	−0.00121	0.000469	0.01
Serine	−0.00347	0.001669	0.04
Choline	−0.00088	0.016049	1.00
Betaine	0.00841	0.003633	0.02
Threonine	0.004593	0.001968	0.02
Methionine	0.001833	0.005933	0.76
Glutamate	0.002866	0.000687	0.0001

*R^2^= 0.44, p = 0.001, AICc = 13.15*.

*AICc, Corrected Aikaike's Information Criterion*.

**Table 3:** Final least squares regression model parameters.

**Term**	**Estimate**	**Std. error**	**Prob > ItI**
**Regression model 2 (*****N*** **=** **67)**			
*MTHFR* 677 T Carrier	−0.0667	0.034556	0.06
*MTHFR* 1298 C Carrier	−0.07195	0.030423	0.02
Glycine	−0.00122	0.000434	0.007
Serine	−0.00344	0.001625	0.04
Betaine	−0.00841	0.003569	0.02
Threonine	0.004801	0.00163	0.005
Glutamate	0.002878	0.000654	<0.0001

*R^2^ = 0.44, p < 0.001 (1.622E-5), AICc = 7.52*.

*AICc, Corrected Aikaike's Information Criterion*.

In the analysis of potential confounding factors, the independent factors based on medication (clozapine or olanzapine use and chlorpromazine equivalents), race, and smoking were not independent predictors of log(HOMA-IR) using the criterion of a *p-*value of >0.1. Although age and BMI were significant by this estimate (each had a *p-*value of < 0.1), none of the potential confounding factors were highly correlated with each other (correlation coefficients smaller than 0.8), one carbon metabolism metabolites or genetic variants, and were left out of the final model to limit the number of independent model factors and maintain power of the model described in Table 3. Results of the regression analysis including potential confounders, and the table of correlation estimates are provided in the Supplementary Material.

## Discussion

The results of this study suggest that in middle-aged patients with schizophrenia, who are stable on an atypical antipsychotic medication regimen, metabolite concentrations and genetic variants within one-carbon metabolism (Figure 1) are capable of predicting log(HOMA-IR) and thus may contribute to the development of CVD. Among the independent factors in our analysis, *MTHFR* variant carriers trended toward higher concentrations of HOMA-IR. Variant carriers have reduced conversion of 5,10-methyl tetrahydrofolate to 5-methyl tetrahydrofolate for subsequent creation of methionine. Despite this association, methionine, which is downstream of *MTHFR*, was not a significant predictor of log(HOMA-IR) in our model which could be attributable to variation in either methionine synthase (*MTR*) expression or activity, neither of which was measured. All metabolites, except threonine and glutamate, were negatively associated with HOMA-IR concentration suggesting that reduced concentrations of one-carbon donors are associated with increased insulin resistance (36, 37). All of these metabolites, except glutamate, are involved in one-carbon metabolism as sources of one-carbon units. Glutamate is also thought to be involved in insulin secretion, which aligns with the observation of increased insulin in this population (38).

### Compounding Data Points to a Role of One-Carbon Metabolism

Our findings support past work that demonstrates a potential link between antipsychotics, anti-oxidant levels, one-carbon metabolism and cardiovascular disease mortality in psychiatric patients (39). Furthermore, changes in one-carbon metabolism are also important for the production of methyl groups for various reactions in the cell including that of DNA methylation. To this end, our group has identified associations between DNA methylation and antipsychotic treatment in more than one tissue as well as the impact of folate (a source of one-carbon) supplementation on gene methylation and some metabolic side effects in patients treated with antipsychotic medications (40–42). Taken together, these findings suggest a strong role for one-carbon metabolism in antipsychotic-induced metabolic side effects and the subsequent increased risk for cardiovascular death in this patient population.

### Glutamate and Inflammatory Pathways

Glutamate, serine, and glycine are required for the synthesis of the antioxidant glutathione within the transsulfuration pathway (43). Glutamate is involved in the rate-limiting step of glutathione synthesis, prior to the addition of glycine (43). In our study, glutamate trended positively with increasing HOMA-IR, whereas glycine and serine concentrations trended negatively with HOMA-IR. This agrees with the recent work of Vangipurapu et al., that identified increased concentrations of glutamate and decreased concentrations of glycine as significantly associated with worsening insulin sensitivity over time in a longitudinal study of ~5,000 men without diabetes at baseline (44). Considering that glutamate is involved in a variety of reactions, including brain-derived neurotrophic factor (45), and elevated serum levels have been associated with a number of inflammatory diseases (46), it is possible that glutamate-derived formation of glutathione within the transsulferation pathway is likely not the key mechanism by which it exerts its role in insulin resistance.

The positive correlation between threonine and HOMA-IR is somewhat discordant with the shared negative relationship between the other metabolites in our predictive model and HOMA-IR. Although threonine is a source of one-carbon groups, it is first metabolized to glycine before entering the folate cycle (Figure 1). Unlike glutamate, variable serum threonine concentrations have not been found to be associated with inflammatory disease states or insulin sensitivity (47).

### Genetic Associations of the Folate Cycle

When considering the contribution of the *MTHFR* genetic variants in our model, we determined that carrier status of either variant trended with higher HOMA-IR values. These results generally agree with other studies in patients with severe mental illness, demonstrating that patients who carry variants that decrease MTHFR enzyme activity have higher rates of metabolic risk factors for CVD (10–12, 18, 48). Our group previously identified that carriers of the *MTHFR* 677 T allele were associated with significantly higher rates of metabolic syndrome diagnosis and that variant carrier status was also associated with increased HOMA-IR value (10, 11). While additional investigators have found that the *MTHFR* 677 T allele was associated with higher rates of metabolic syndrome (12), other studies have found a lack of association with the *MTHFR* 677 T allele (9, 48). In some instances when both variants were studied, there was a lack of association with the *MTHFR* 677 T variant, but the *MTHFR* 1298 C variant (or CC homozygote) was associated with metabolic syndrome prevalence (48) or the development of worse metabolic outcomes following 3 months of atypical antipsychotic treatment (18). Cumulatively, results from our group and others demonstrate that variants within the *MTHFR* gene known to cause reduced enzyme function are associated with higher risk of cardiovascular disease.

## Limitations

We acknowledge that there are several limitations of our study. As a pilot investigation with a small sample size, we recognize that this model will need to be tested in larger, independent populations to validate the ability of metabolomics and genetic variants within one-carbon metabolism to predict HOMA-IR. Nevertheless, despite this limitation, our study cohort consisted of well-characterized schizophrenia patients on long-term antipsychotic therapy with targeted profiling of both genetic and metabolite factors in the one-carbon pathway. Another limitation of our study that may impact external validity is the variable past and current medication exposure. Meta-analyses have demonstrated that CVD risk increases after the first exposure to antipsychotics when compared to similar treatment naïve patients (6), and that olanzapine and clozapine are known to be associated with higher risk of metabolic complications (34). Our model did not identify a relationship between current medication use and HOMA-IR, which may be due to the cross-sectional nature of the study and inability to associate medication changes with metabolite variability. In future studies it will be important to test associations between genetic variants, metabolites, and medication use over time to assess whether these relationships are consistent depending on extent and type of medication exposure. It will also be important to consider, in larger samples sizes, additional pharmacogenetic markers that are associated with antipsychotic outcomes, as well as serum medication concentrations. As an example, select variants in CYP1A2 are known to lead to lower-than-anticipated serum concentrations of clozapine and greater risk of treatment failure in the presence of enzyme inducers (49). Finally, the cross-sectional nature of this study limits drawing any conclusions about the impact of environmental factors over time, such as prior smoking and how that relates to the ability of metabolite concentrations, in concert with genetic data, to predict HOMA-IR concentrations.

## Conclusions

In summary, a least squares regression model including metabolite concentration data of primarily one-carbon unit donors within one-carbon metabolism, and genetic variants in *MTHFR*, are able to predict HOMA-IR. This is clinically important as elevated HOMA-IR has been associated with increased risk of developing diabetes, and future cardiovascular disease. Cumulatively, these results should be interrogated further in larger, prospective studies to determine precisely how variations in this complex metabolic and genetic network relate to mechanisms underlying the development of CVD risk in patients with severe mental illness, such as schizophrenia, or in patients who are otherwise healthy. Ideally, future precision health research will work toward leveraging an understanding of genetic and environmental markers of metabolism to identify opportunities for tailoring antipsychotic prescribing, or supplementation with adjunctive therapies, to decrease the risk of metabolic side effects in patients with serious mental illness.

## Data Availability Statement

The genetic datasets presented in this article are not readily available because they contain protected health information. Requests to access the datasets should be directed to Dr. Vicki Ellingrod. The metabolomics data used in this study are accessible through the Metabolomics Workbench: https://www.metabolomicsworkbench.org/.

## Ethics Statement

The studies involving human participants were reviewed and approved by The Study Protocol received Institutional Review Board (IRB) approval from the University of Michigan (IRBMED HUM00017774) and the following local organizations: the Detroit-Wayne County Community Mental Health Agency (DWCCMHA), Washtenaw County Health Organization (WCHO), and the Ann Arbor Veterans Affairs Medical Center. The patients/participants provided their written informed consent to participate in this study.

## Author Contributions

KW, VE, AZK, and KB contributed to the study design. KW, KB, AJ, CM, LY, AK, and KS contributed to the metabolomics and genomics analyses. KW, KB, and AJ drafted the initial manuscript. All authors contributed to critical revision of the manuscript.

## Conflict of Interest

The authors declare that the research was conducted in the absence of any commercial or financial relationships that could be construed as a potential conflict of interest.
