# Supplementary material for: Genetic and Metabolite Variability in One-Carbon Metabolism Applied to an Insulin Resistance Model in Patients With Schizophrenia Receiving Atypical Antipsychotics
Source: Front Psychiatry. 2021 May 25;12:623143. doi: 10.3389/fpsyt.2021.623143 (PMC8185170; doi:10.3389/fpsyt.2021.623143)
Supplement: Supplementary file 1 [file Data_Sheet_1.docx]

Supplementary Material

| **Regression Model (N=67)** | | | |
| --- | --- | --- | --- |
| ***Term*** | ***Estimate*** | ***Std Error*** | ***Prob>ItI*** |
| *MTHFR* 677 T Carrier | -0.06037 | 0.030862 | 0.0567 |
| *MTHFR* 1298 C Carrier | -0.05531 | 0.026944 | 0.046 |
| Serine | -0.00237 | 0.001337 | 0.0829 |
| Glycine | -0.00102 | 0.000393 | 0.0131 |
| Betaine | -0.00771 | 0.003541 | 0.0348 |
| Threonine | 0.002953 | 0.00176 | 0.1004 |
| Glutamate | 0.001078 | 0.000674 | 0.1168 |
| Age | 0.012122 | 0.003962 | 0.0037 |
| Chlorpromazine equivalents, atypical antipsychotics only | -0.0001 | 7.82E-05 | 0.1983 |
| Waist circumference | -0.0618 | 0.082517 | 0.4578 |
| Race (Caucasian/other) | 0.015071 | 0.030927 | 0.6284 |
| Smoker | -0.02137 | 0.026001 | 0.4155 |
| BMI | 0.022593 | 0.004595 | <.0001 |
| Clozapine/olanzapine use | -0.03079 | 0.028085 | 0.2788 |
| *R^2^= 0.69, p=6.601E-9 , AICc= 13.84* | | | |
| **Supplemental Table 1:** Least squares regression model parameters for initial input including all detected metabolites within one-carbon metabolism and potential confounders  BMI: body mass index; AICc: Corrected Aikaike’s Information Criterion | | | |

|  |  |  |  |  |  |  |  |  |  |  |  |  |  |
| --- | --- | --- | --- | --- | --- | --- | --- | --- | --- | --- | --- | --- | --- |

|  |  |  |  |  |  |  |  |  |  |
| --- | --- | --- | --- | --- | --- | --- | --- | --- | --- |
| **Correlation of Estimates** | | | | | | | | | |
| ***Independent variable*** | ***Intercept*** | ***MTHFR 677 T carrier*** | ***MTHFR 1298 C carrier*** | ***Serine*** | ***Glycine*** | ***Betaine*** | ***Threonine*** | ***Glutamate*** | ***Age*** |
| Intercept | 1 | -0.146 | -0.0564 | -0.2609 | 0.0666 | -0.1431 | 0.0087 | 0.2323 | -0.7106 |
| MTHFR 677 T carrier | -0.146 | 1 | 0.1651 | -0.1044 | 0.0801 | -0.0092 | 0.0794 | -0.0681 | 0.0763 |
| MTHFR 1298 C carrier | -0.0564 | 0.1651 | 1 | 0.2064 | 0.1246 | -0.0197 | -0.0315 | 0.0078 | -0.2375 |
| Serine | -0.2609 | -0.1044 | 0.2064 | 1 | -0.1268 | 0.0909 | -0.3299 | -0.1196 | -0.0675 |
| Glycine | 0.0666 | 0.0801 | 0.1246 | -0.1268 | 1 | -0.0582 | -0.2724 | -0.1452 | -0.2766 |
| Betaine | -0.1431 | -0.0092 | -0.0197 | 0.0909 | -0.0582 | 1 | -0.4876 | -0.2849 | -0.0627 |
| Threonine | 0.0087 | 0.0794 | -0.0315 | -0.3299 | -0.2724 | -0.4876 | 1 | 0.4252 | 0.0102 |
| Glutamate | 0.2323 | -0.0681 | 0.0078 | -0.1196 | -0.1452 | -0.2849 | 0.4252 | 1 | -0.1717 |
| Age | -0.7106 | 0.0763 | -0.2375 | -0.0675 | -0.2766 | -0.0627 | 0.0102 | -0.1717 | 1 |
| Chlorpromazine equivalents, atypical antipsychotics only | -0.2334 | 0.0659 | 0.1494 | 0.1056 | -0.0053 | 0.2351 | -0.24 | -0.1993 | 0.0051 |
| Waist circumference | 0.2587 | -0.0769 | -0.0157 | 0.0268 | -0.0329 | 0.3681 | -0.1957 | 0.0754 | -0.2463 |
| Race (Caucasian/other) | 0.0085 | -0.2806 | -0.1948 | 0.0551 | 0.0698 | 0.2653 | -0.3045 | -0.1799 | 0.0883 |
| Smoker | -0.1054 | 0.1823 | 0.0195 | 0.0974 | -0.0268 | 0.12 | -0.0895 | -0.1378 | -0.0078 |
| BMI | -0.6247 | 0.0415 | 0.1568 | 0.1605 | 0.1368 | 0.0243 | -0.1951 | -0.4921 | 0.2638 |
| Clozapine/Olanzapine use | 0.0932 | 0.168 | 0.006 | -0.038 | -0.0134 | -0.1575 | 0.1703 | 0.113 | -0.0386 |
| **Supplemental Table 2:** Correlation estimates for model parameters described in Supplemental Table 1 | | | | | | | | | |

| **Correlation of Estimates** | | | | |  |  |
| --- | --- | --- | --- | --- | --- | --- |
| ***Independent variable*** | ***Chlorpromazine equivalents, atypical antipsychotics only*** | ***Waist circumference*** | ***Race (Caucasian/other)*** | ***Smoker*** | ***BMI*** | ***Clozapine/ Olanzapine use*** |
| Intercept | -0.2334 | 0.2587 | 0.0085 | -0.1054 | -0.6247 | 0.0932 |
| MTHFR 677 T carrier | 0.0659 | -0.0769 | -0.2806 | 0.1823 | 0.0415 | 0.168 |
| MTHFR 1298 C carrier | 0.1494 | -0.0157 | -0.1948 | 0.0195 | 0.1568 | 0.006 |
| Serine | 0.1056 | 0.0268 | 0.0551 | 0.0974 | 0.1605 | -0.038 |
| Glycine | -0.0053 | -0.0329 | 0.0698 | -0.0268 | 0.1368 | -0.0134 |
| Betaine | 0.2351 | 0.3681 | 0.2653 | 0.12 | 0.0243 | -0.1575 |
| Threonine | -0.24 | -0.1957 | -0.3045 | -0.0895 | -0.1951 | 0.1703 |
| Glutamate | -0.1993 | 0.0754 | -0.1799 | -0.1378 | -0.4921 | 0.113 |
| Age | 0.0051 | -0.2463 | 0.0883 | -0.0078 | 0.2638 | -0.0386 |
| Chlorpromazine equivalents, atypical antipsychotics only | 1 | -0.0492 | -0.1408 | 0.1627 | 0.1142 | -0.1288 |
| Waist circumference | -0.0492 | 1 | 0.391 | -0.2148 | -0.5516 | -0.1897 |
| Race (Caucasian/other) | -0.1408 | 0.391 | 1 | -0.0953 | -0.072 | -0.1328 |
| Smoker | 0.1627 | -0.2148 | -0.0953 | 1 | 0.2046 | 0.1574 |
| BMI | 0.1142 | -0.5516 | -0.072 | 0.2046 | 1 | 0.0131 |
| Clozapine/Olanzapine use | -0.1288 | -0.1897 | -0.1328 | 0.1574 | 0.0131 | 1 |
| **Supplemental Table 2, cont.** | | | | |  |  |
